# Supplementary figures and images for: Different Approximation Methods for Calculation of Integrated Information Coefficient in the Brain during Instrumental Learning
Source: Brain Sci. 2022 May 3;12(5):596. doi: 10.3390/brainsci12050596 (PMC9138974; doi:10.3390/brainsci12050596)

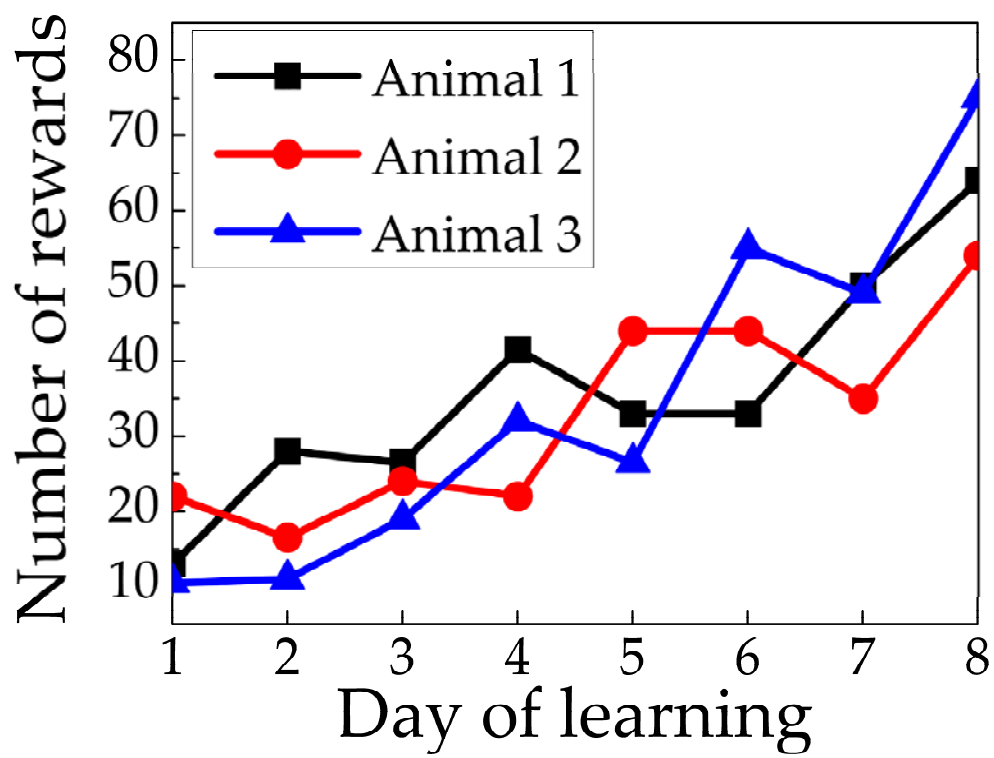

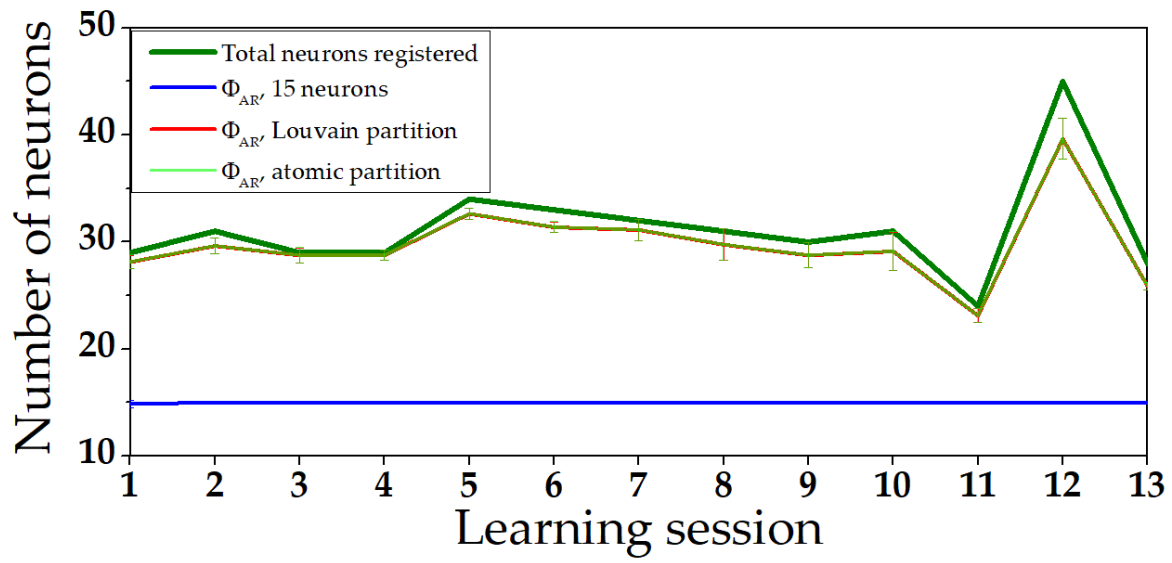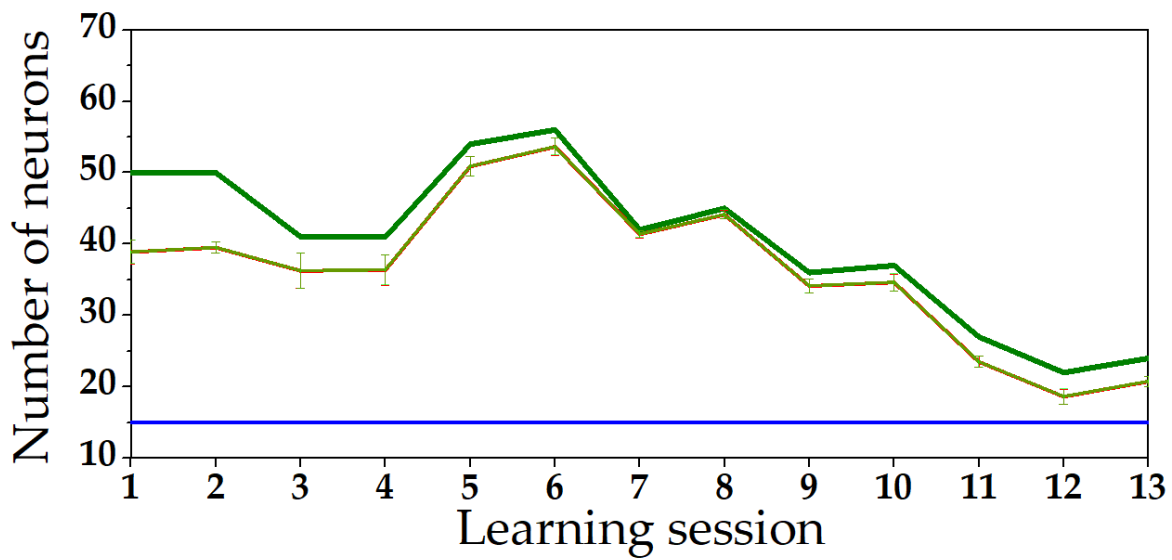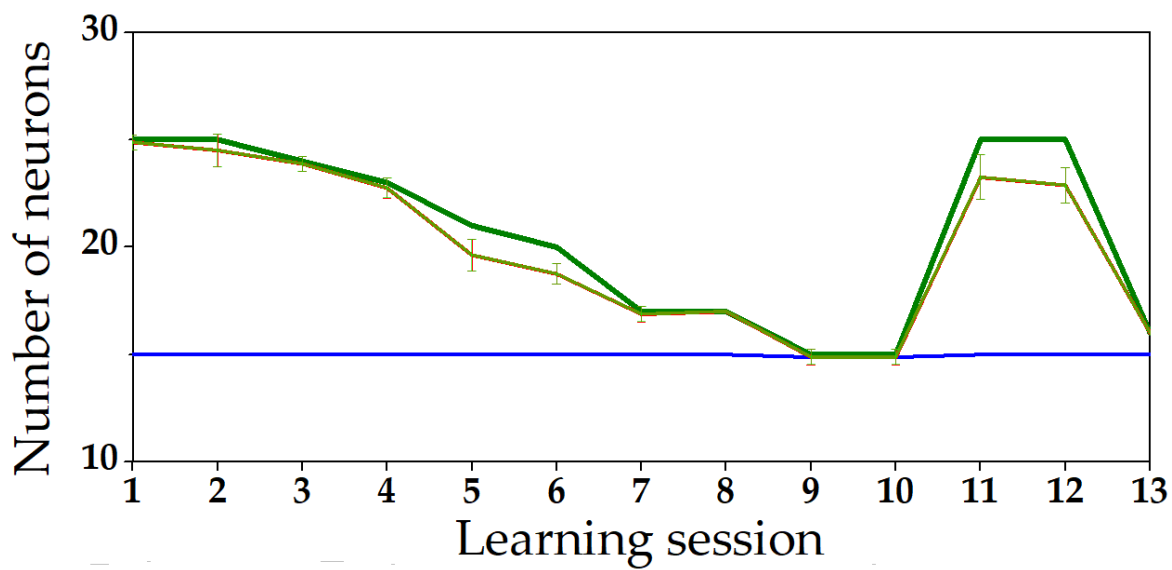

Supplement: Supplementary file 1 [file brainsci-12-00596-s001.zip › brainsci-1632803-supplementary.pdf]
